# A post-trial survey to assess the impact of dissemination of results and unmasking on participants in a 13-year randomised controlled trial on age-related cataract

**DOI:** 10.1186/1745-6215-12-148

**Published:** 2011-06-14

**Authors:** Sally L Williams, Luigina Ferrigno, Giovanni Maraini, Francesco Rosmini, Robert D Sperduto

**Affiliations:** 1 Sezione di Oftalmologia, Università degli Studi di Parma, Via Gramsci 14, 43126 Parma, Italy; 2 Laboratorio di Epidemiologia e Biostatistica, Istituto Superiore di Sanità, Roma, Italy; 3 National Eye Institute, Bethesda, MD, USA

## Abstract

**Background:**

The Italian-American Clinical Trial of Nutritional Supplements and Age-Related Cataract was designed to assess the impact of a multivitamin-mineral supplement on age-related cataract. Trial results showed evidence of a beneficial effect of the supplement on all types of cataract combined, opposite effects on two of the three types of cataract (beneficial for nuclear opacities and harmful for posterior sub-capsular opacities) and no statistically significant effect on cortical opacities. No treatment recommendations were made. A post-trial survey was conducted on 817 surviving elderly participants to assess their satisfaction, their understanding of treatment assignment to supplement or placebo and the success of masking.

**Methods:**

Trial results were communicated by letter and the level of satisfaction and of understanding of the results was assessed by a questionnaire. Participants were offered the option of being unmasked: a second questionnaire was administered to this subset to assess their understanding of the randomisation process and the success of masking.

**Results:**

610 participants (74.7%) responded to the survey:

94.6% thought the description of the results was "very clear" or "quite clear", 5.4% "not clear" or "do not know"; 89.8% considered the results "very interesting" or "quite interesting", 10.2% "not interesting" or "do not know"; 60.3% expressed "satisfaction", 17.2% "both satisfaction and concern", 2.6% "concern", 19.9% "indifference" or "do not know".

480 participants (78.7%) accepted the offer to be unmasked to their treatment assignment: 395 (82.3%) recalled/understood the possibility of assignment to vitamins or placebo, 85 (17.7%) did not. 68 participants (17.2%) thought they had taken vitamins (79.4% were correct; p = 0.0006), 47 (11.9%) thought they had taken placebo (59.6% were correct; p = 0.46) and 280 (70.9%) declared they did not know.

**Conclusions:**

The results were made difficult to explain to study participants by the qualitatively different effect of treatment on the two most visually significant types of cataract. Although the study did not lead to a recommendation to use the dietary supplement, the vast majority of participants reported satisfaction after they received the results but almost 20% of the participants expressed some concern. Masking to treatment assignment was successful in the majority of participants.

## Background

The Italian-American Clinical Trial of Nutritional Supplements and Age-Related Cataract (CTNS) is a 13-year individually-randomised, double-masked, single-centre, placebo-controlled trial designed to investigate whether adding a daily multivitamin-mineral supplement to the diet of a generally well-nourished, supplement-naïve population could affect the onset or progression of age-related cataract [1]. One thousand and twenty residents of Parma, Italy, aged 55 to 75 years and with no or early cataract, were randomly assigned to treatment with a daily multivitamin-mineral tablet or a placebo. Participants were treated and followed for an average of 9.0 +/- 2.4 years, from January 1996 to May 2007, with few losses to follow-up and excellent compliance with medication assignment, as assessed by pill count and vitamin plasma levels. Participation involved 2 baseline visits and a maximum of 20 follow-up visits at six month intervals that included: eye examination with visual acuity measurement, pupillary dilation and lens photography, anthropometric measurements, interviews and blood collections.

Use of the dietary supplement appeared to have a beneficial effect on any cataract (all types of cataract combined) and on pure nuclear cataract; a harmful effect was observed on posterior sub-capsular cataract and no statistically significant effect was observed on cortical cataract and on important functional end-points (visual acuity or cataract surgery). These findings, in particular the opposite effects on the two most visually significant cataract types, prevented us from making recommendations about use of one-a-day multivitamin-mineral supplements to affect the risk of cataract development [2]. The Steering Committee unanimously decided to share the results with CTNS participants even though they were difficult to convey and there was some risk of generating misunderstandings. Communicating the results of a study to participants is a generally accepted practice in clinical trials [3], but sometimes participants do not wish to know the results [4,5] nor to be unmasked to their treatment assignment and may even react negatively [6]. Little is known about the opinions and expectations of participants on sharing the results of a study such as CTNS, which involved an intervention with essentially no side effects and a condition (cataract) that could be effectively treated with surgical intervention. This paper describes the methodology used to share the results with CTNS participants and the findings of a satisfaction survey administered at the end of the study.

## Methods

The last CTNS follow-up visit occurred in May 2007. A manuscript describing the study results was submitted in November 2007 and published in April 2008 [2]. The material for dissemination of the results to the participants (a letter and two questionnaires) was prepared by the Steering Committee. The letter on behalf of the principal investigator and the study manager thanked participants for their participation in the trial and included a lay summary of trial results and conclusions. Given the age of our patients and the objective of our survey, we chose to use a questionnaire with a very simple structure, easy to complete and patient-friendly. The questionnaires used in the survey were not formally validated but the letter and enclosed questionnaire were shown to a small number of elderly patients to ensure that they were clear and comprehensible (see Additional file 1 and Additional file 2). The material was approved by the Ethics Committee in Parma on April 10, 2008 and dissemination of results and questionnaires to surviving CTNS participants began in mid-April 2008 and completion of questionnaires continued until July 2009. The study manager and an interviewer were responsible for maintaining contact with the patients. The letter and questionnaire 1 were initially mailed with a pre-paid return envelope to 50 CTNS participants: since the questionnaire seemed to be sufficiently clear to these patients, we then proceeded with the dissemination of the results. Letters were sent in batches until September 2008 to spread the workload generated by the letters. The questionnaire included 8 closed questions designed to evaluate participants' level of satisfaction and understanding of the results and an offer to be informed about individual treatment assignment. Participants were invited to contact study staff if they required further clarifications.

Upon receipt of a completed questionnaire, the study manager telephoned the participants who indicated they wanted to know whether they had been assigned the vitamin-mineral supplement or the placebo. Patients were offered the option of receiving this information on the telephone or face-to-face at the clinic. In the latter case they were offered an eye examination. During the telephone contact or face-to-face at the clinic, the study manager administered a second brief questionnaire (see Additional file 3) and evaluated the understanding of the randomisation process and the success of masking by discussing with the participants whether they thought they had taken the supplement or the placebo.

The study manager, with the help of an interviewer, telephoned all participants who did not return the questionnaire: in some instances the letter and questionnaire were resent to the participants, but generally participants who had not responded to the questionnaire preferred to be informed about the results directly by the study manager or by one of the study ophthalmologists during a scheduled visit at the clinic. In this case both questionnaires were administered to the participant by the study manager.

Analyses were carried out using the STATA statistical package. Student's t test was used for assessing differences between means and Chi-squared test for differences between proportions.

To assess whether participants had recognised their treatment assignment, we compared the observed distribution with a hypothetical distribution of equally likely answers (p = 0.5) using a χ^2 ^goodness of fit test.

## Results

**Figure 1:**
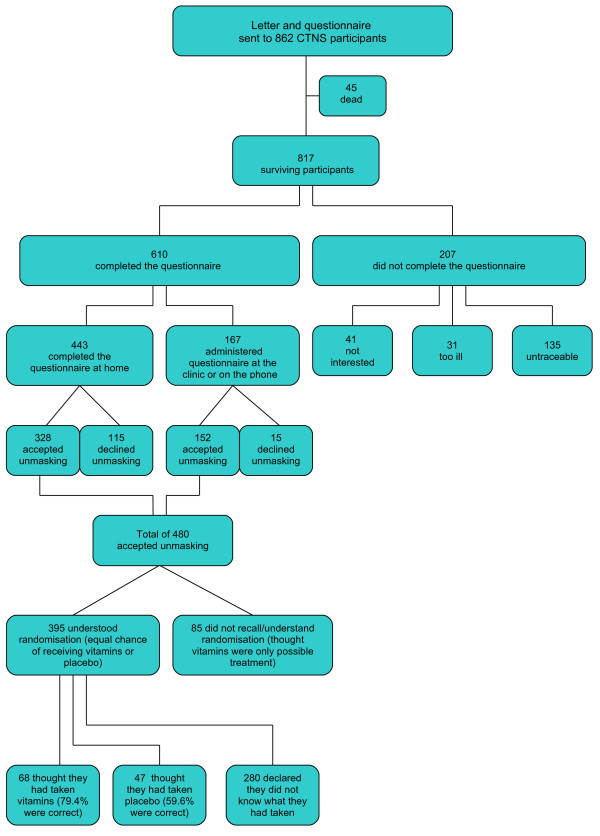
**Flow diagram**.

The letter describing the results of the trial was sent to the 862 CTNS participants (average age 80.5 years, range 68-89) who were alive at the end of the clinical trial (see Figure 1). The frequency figures in Table 1 were used to demonstrate the effect of treatment with the dietary supplement on age-related cataract. Participants were told that the overall effect of the supplement on the three types of cataract (nuclear, cortical, posterior sub-capsular), when considered as a single disease, was beneficial but when the opacities were considered separately, a beneficial effect was noted for nuclear opacities, an increase in risk was observed for posterior sub-capsular opacities and no statistically significant effect was observed for cortical opacities. Furthermore it was specified that from a clinical viewpoint no relevant differences between treatment groups were detected in visual function performance or in the number of cataract surgeries. The letter indicated that, because of the qualitatively different effect of treatment on the different types of cataract, the investigators could not recommend regular use of the supplement for the prevention of cataract.

**Table 1:** Main results of the Italian-American clinical trial of nutritional supplements and age-related cataract, Parma, 1996-2008

	RESULTS PROVIDED TO PARTICIPANTS FREQUENCY OF EVENTS		**PUBLISHED RESULTS**[2] ASSOCIATION BETWEEN TREATMENT AND CATARACT EVENT	
	
LENS EVENT	Treatment in 510 participants	Placebo in 510 participants		
	
	**N**.	**N**.	Hazard Ratio*	95% Confidence Interval
Cortical	96	118	0.78	0.60 - 1.02

Nuclear	84	118	0.66	0.50 - 0.88

PSC**	74	37	2.00	1.35 - 2.98

Cataract surgery	88	90	0.95	0.71 - 1.28

Any lens event***	227	255	0.82	0.68 - 0.98

* Adjusted for age and type of opacity

** Posterior Sub-Capsular

*** Participants could have more than one event

Forty-five of the 862 participants (5.2%) to whom the results were sent died after the end of the study before receiving the results. Eight hundred and seventeen participants (94.8%) were alive at the time of the post trial survey as verified in the National Health Service local database.

Six hundred and ten of the 817 surviving participants (74.7%) responded to the survey: of these 443 (72.6%) completed the mailed questionnaire at home and 167 (27.4%) were administered the questionnaire at the clinical centre during a scheduled visit or on the telephone.

Two hundred and seven subjects did not respond (25.3%): 72 refused or were unable to complete the questionnaire (41 not interested, 31 too ill) and 135 could not be contacted (inactive telephone number, closed envelopes with results returned to clinic). Non-responders were more likely than responders to be older (p < 0.001), female (p = 0.002), unmarried (p = 0.002), and to have undergone cataract surgery (p = 0.03) (Table 2).

**Table 2:** Characteristics of participants by response to dissemination of results and by acceptance of treatment unmasking

	SURVEY			TREATMENT UNMASKING		
CHARACTERISTICS	Responders (n = 610)	Non-responders (n = 207)	**χ**^**2 **^**test**	Accepted (n = 480)	Declined (n = 130)	**χ**^**2 **^**test**
	%	%	P	%	%	P
SEX						
Male	53.3	41.1		51.5	60.0	
Female	46.7	58.9	0.002	48.5	40.0	0.08

MARITAL STATUS						
Married	78.9	67.1		78.7	79.2	
Other	21.1	32.9	0.002	21.3	20.8	0.81

EDUCATION						
Primary school	44.9	54.6		42.3	54.6	
Middle school	25.8	21.3		28.1	16.9	
High school	29.3	24.1	0.06	29.6	28.5	0.01

TREATMENT ASSIGNED						
Placebo	48.8	51.7		46.5	57.7	
Treatment	51.2	48.3	0.48	53.5	42.3	0.02

CATARACT SURGERY						
Yes	21.2	28.5		21.5	20.0	
No	78.8	71.5	0.03	78.5	80.0	0.72

AGE (MEAN)						
Years	79.7	82.1	<0.001*	79.5	80.4	0.04*

* T test

Overall 63.3% of the responders judged the description of the results "very clear", 31.3% "quite clear", 5.4% "unclear" or "do not know"; 46.4% of the responders judged the results "very interesting", 43.4% "quite interesting", 10.2% not interesting or "do not know" (Table 3). If patients were subdivided according to selected mode of communication with study staff (face-to-face, telephone, no contact), the results were considered "very interesting" by 52.4% of patients who attended a scheduled visit at the clinical centre, by 47.4% of those who communicated with study staff on the telephone and by 35.4% of those who completed and returned the questionnaire without requesting to communicate with study staff (p = 0.01). After acknowledging the results 60.3% of the responders expressed "satisfaction", 17.2% "both satisfaction and concern", 2.6% "concern", 19.9% "indifference" or "do not know".

**Table 3:** Main responses to the post-trial questionnaire 1

QUESTIONS		N.	%
BEFORE DISCLOSURE			
Do you think the description of the results of the study is clear?	Very clear	386	63.3
	Quite clear	191	31.3
	Not clear	9	1.5
	Do not know	24	3.9
	TOTAL	610	100.0
			
Do you think the results of the study are interesting?	Very interesting	283	46.4
	Quite interesting	265	43.4
	Not interesting	16	2.6
	Do not know	46	7.6
	TOTAL	610	100.0
			
What did you feel when you learned about the results of the study?	Satisfaction	368	60.3
	Concern	16	2.6
	Both satisfaction and concern	105	17.2
	Indifference	68	11.2
	Do not know	53	8.7
	TOTAL	610	100.0
			
Do you think it is appropriate to receive the results of the study by letter?	Very appropriate	453	74.3
	Quite appropriate	117	19.2
	Not appropriate	16	2.6
	Do not know	24	3.9
	TOTAL	610	100.0
			
Would you recommend to other persons to take part in a study like CTNS?	Yes, definitely	554	90.8
	Yes, probably	40	6.6
	Probably not	4	0.6
	Definitely not	1	0.2
	Do not know	11	1.8
	TOTAL	610	100.0
AFTER DISCLOSURE			
Now that you know what treatment you took would you recommend to other persons to take part in a similar study?	Yes, definitely	375	94.9
	Yes, probably	16	4.1
	Probably not	3	0.8
	Do not know	1	0.2
	TOTAL	395	100.0

To receive the results by mail was deemed "very appropriate" by 74.3% of the responders and "quite appropriate" by 19.2%, "not appropriate" or "do not know" by 6.5%. However 299 (49%) of the responders requested further clarifications from the study staff, e.g. what type of cataract they had and how the trial conclusions affected them as individuals.

The offer to reveal treatment assignment was accepted by 480 of 610 (78.7%) responders. A total of 289 (60.2%) participants chose to receive this personal information on the telephone, 191 (39.8%) at the clinic during a scheduled visit. Eighty-five responders (17.7%), according to the study manager's evaluation, did not recollect or understand that assignment could be to one of two interventions since they believed that the dietary supplement was the only treatment offered. Of the 395 responders who clearly understood that assignment could be to either treatment, 68 (17.2%) thought they had been assigned to the vitamin mineral arm (79.4% were correct; χ^2 ^goodness of fit test: p = 0.0006), 47 (11.9%) to the placebo arm (59.6% were correct; χ^2 ^goodness of fit test: p = 0.46), 280 (70.9%) did not know. The subjects who were not interested in being unmasked were older (p = 0.04), had a lower level of education (p = 0.01) and were assigned more frequently to placebo (p = 0.02) (Table 2). After completion of the unmasking process, the participants who had been assigned to placebo, when asked "would you recommend to other persons to take part in a study like CTNS ?", answered: "definitely yes" (95.6%) and "probably yes" (4.4%); while those assigned to the vitamin/mineral supplement answered "definitely yes" (94.3%) and "probably yes" (3.8%). The satisfaction rating after unmasking was similar to that prior to unmasking.

## Discussion

The World Medical Association Declaration of Helsinki (October 2008) states that "at the conclusion of the study, patients entered into the study are entitled to be informed about the outcome of the study and to share any benefits that result from it, for example, access to interventions identified as beneficial in the study or to other appropriate care or benefits" [7].

While participation in clinical studies is sometimes motivated mainly by altruism [8], most investigators claim that it is important to inform participants about the results of clinical trials so that participants are not treated as mere means to ends and to fulfil the ethical obligation of respect for human dignity [9]. Although there are no provisions in Italian law on whether and how to provide study results, some investigators recognise the need to communicate the therapeutic [6,8] or preventative [10,11] findings of a study to participants, even in studies such as CTNS which did not result in any preventative or therapeutic recommendations [12]. Giving the participants the opportunity to understand the contribution of the study to scientific knowledge can be a way of highlighting the central role that they played in the research project [13].

A year elapsed from the last follow-up visit to publication of the trial results in a peer-reviewed journal. Moreover the advanced age of the participants and difficulties in finding participants who had changed their addresses and telephone numbers complicated and delayed dissemination of results. This caused some CTNS participants to complain about the delay in receiving the results. It is generally accepted that results should be offered promptly [9] and that it is critical to maintain contacts with the participants until provision of results [14].

Efforts were made to present data in the letter so that they were understandable to a lay population. For example, instead of presenting hazard ratios and tests of significance as was done in the published paper, absolute frequencies of end-points in the study arms were presented. Interpreting the frequencies was made easier because the same number of participants was randomised to each arm of the study. About 94% of participants judged the presentation of results to be "very clear" or "quite clear." Only slightly fewer participants found the results to be "very interesting" or "quite interesting." Although the results had no impact on clinical practice, the positive public image of the study did not seem to be affected. Almost all participants indicated they would recommend that others take part in a study such as CTNS. It is possible that, since 443 post-trial questionnaires were completed at home by the participants and 167 were administered to participants at the clinic, some elements of bias may have been introduced in participants' responses to post-trial questionnaire 1.

Concerns have been raised that participants in trials could be stressed [6] or even harmed [8,9] by provision of personal and/or of general study results [15]. Possible emotional harmful effects include: reliving difficult moments, learning of being part of the "inferior" trial arm or being at higher risk for a health-related problem [12]. The risk of such harmful effects was not great in CTNS because an effective surgical remedy is available for age-related cataract and the intervention is known to have essentially no side effects. However, the results of CTNS could have been cause of concern because of the increase in posterior sub-capsular opacities that was unexpectedly noted among those in the active treatment arm. In fact almost 20% of the participants expressed some concern: 2.6% of the participants answered that they experienced concern after receiving the results and 17.2% declared that they felt "both satisfaction and concern".

In order to respect the right of participants not to know their treatment assignment, this information was provided only to those who answered affirmatively to our offer [15,16]. The participants who did not wish to be informed about their assignment represent a self-selected group to whom the second questionnaire was not administered. Since no information was collected on their understanding of the randomisation process and on the success of masking, some element of selection bias may have been introduced for responses to the second questionnaire. Participants assigned to placebo were significantly over-represented in the group who declined unmasking (58% vs. 46%). The study tablets were manufactured to appear indistinguishable, and adherence to treatment assignment was similar in the two arms over the course of the trial. However the higher proportion of participants in the vitamin-mineral arm who correctly identified their assigned treatment (79.4%; χ^2 ^goodness of fit test: p = 0.0006) seems to suggest that they may have recognised the supplement perhaps due to the aroma or the taste of the tablets.

Being informed of their treatment assignment did not seem to discourage participants in the placebo arm, most of whom indicated that they would have certainly recommended participation in a similar trial to other people.

We recognise the limitations of our post-trial survey, which, by design, is subject to potential bias. We cannot exclude the possibility that differences in the characteristics of responders and non-responders (see age, sex and lower education in Table 2) may have affected the findings for our study population. Potential biases may have been introduced by the questionnaires which were not validated and did not use Likert scales.

The methods we used in communicating results from the study and our findings may not be generalisable to many other studies because of some unique features of our trial. We dealt with a non life-threatening condition (cataract) for which there is an effective treatment and a possible intervention (one-a-day vitamin) which had essentially no adverse effects. In studies of more serious conditions or studies that result in treatment recommendations it may be necessary to disseminate the results in a more timely fashion through immediate direct contacts or group presentations. However, regardless of the nature of the study there is now general recognition that investigators have an obligation to share the results of studies with participants.

## Conclusions

Dissemination of trial results is a process that should be tailored to the context of each trial. It depends on the condition of interest, on the population, on the interventions and on the outcomes, in particular on the adverse events and on the trial results. In this paper we have presented our experience in an elderly population, affected by age-related cataract (a non-life threatening disease), randomised to a one-a-day vitamin/mineral supplement or placebo with few adverse events. The trial results were not easy to communicate because of the beneficial effect of supplement use on the primary end-point of the study, the progression of any type of opacity or cataract surgery, and the statistically significant, but opposite, effects of supplement use on the two most visually significant opacity subtypes. In general, participants reported satisfaction with how the results were disseminated and interest in the findings.

## List of abbreviations

CTNS: Clinical Trial of Nutritional Supplements, acronym for "The Italian-American Clinical Trial of Nutritional Supplements and Age-Related Cataract".

## Competing interests

The authors declare that they have no competing interests.

## Authors' contributions

SLW designed the study, collected data and contributed to manuscript preparation, LF contributed to study design, manuscript preparation and performed the statistical analysis, GM was involved in interpretation of the results, manuscript preparation and critical revision, FR contributed to study design, manuscript preparation and critical revision, RDS was involved in manuscript preparation and substantive critical review. All authors read and approved the final manuscript.

## Supplementary Material

Additional file 1 **Letter to CTNS participants**. Letter with trial results sent to CTNS participants. Click here for file

Additional file 2 **Post-trial questionnaire 1**. Questionnaire to assess patient satisfaction and understanding of the results. Click here for file

Additional file 3 **Post-trial questionnaire 2**. Questionnaire to assess patient understanding of treatment assignment to supplement or placebo and the success of masking. Click here for file
