# Supplementary material for: A post-trial survey to assess the impact of dissemination of results and unmasking on participants in a 13-year randomised controlled trial on age-related cataract
Source: Trials. 2011 Jun 14;12:148. doi: 10.1186/1745-6215-12-148 (PMC3136405; doi:10.1186/1745-6215-12-148)
Supplement: Additional file 1 — Letter to CTNS participants. Letter with trial results sent to CTNS participants. [file 1745-6215-12-148-S1.DOC]

**LETTER TO CTNS PARTICIPANTS**

Dear …….,

Now that the “Italian American Study of the effect of vitamins and minerals on age-related cataract” (Studio Italia-USA sulla Cataratta Senile), hereafter denoted as CTNS, has been completed, we wish to inform you of the results of the study and to take the opportunity to thank you for your commitment and collaboration as a participant for so many years.

As you know, CTNS began in November 1995 and ended in May 2007. The CTNS investigators have completed the analysis of the data and the results are published in the April issue of “Ophthalmology”, the scientific journal of the Academy of Ophthalmology of the United States of America.

The objective of the study was to assess if a regular intake of vitamins and minerals at the recommended daily dosage can prevent age-related cataract or slow its development.

1020 persons took part in the study: at the beginning of the study half of the participants (510 persons) were assigned to treatment with vitamin/minerals and the other half (510 persons) were assigned to a placebo (an inactive pill with no effect). Neither the participants nor the investigators in Parma knew who was taking what, to guarantee the maximum objectivity in data collection.

The results of the study can be summarized as follows:

1. among the participants who took vitamin/minerals, 227 cataracts occurred or progressed (considering all three types of cataract: nuclear, cortical and posterior sub-capsular) whereas, among the participants who took placebo, 255 cataracts occurred or progressed. In other words, cataract was less frequent among the participants who took vitamin/minerals;

If we consider the three types of cataract one by one (nuclear, cortical and posterior sub-capsular) the results show that:

2. among the participants on vitamin/minerals, 84 nuclear cataracts occurred (or progressed) whereas, among the participants on placebo, 118 nuclear cataracts occurred (or progressed). In other words, nuclear cataract was less frequent among the participants who took vitamin/minerals;

3. among the participants on vitamin/minerals, 96 cortical cataracts occurred (or progressed) whereas, among the participants on placebo, 118 cataracts occurred (or progressed). In other words, cortical cataract was less frequent among the participants who took vitamin/minerals;

4. among the participants who took vitamin/minerals, 74 posterior sub-capsular cataracts occurred (or progressed) whereas, among the participants who took placebo, 37 cataracts occurred (or progressed). In other words, posterior sub-capsular cataract was more frequent among participants who took vitamin/minerals;

5. the number of participants with a decrease in vision (defined as a 15 letter or more loss in vision from the beginning of the study) was 144 among the participants who took vitamin/minerals and 139 among the participants who took placebo, a difference too small to be considered relevant;

6. 88 participants taking vitamin/minerals and 90 participants taking placebo had cataract surgery, a difference too small to be considered relevant;

7. No difference in side effects was observed in participants taking vitamin/minerals compared with participants taking placebo.

**Conclusions**

Overall there was a decrease in the development of cataract, in particular nuclear cataract, in participants who took vitamin/mineral tablets. However, it is necessary to take into account the opposite effect observed on posterior sub-capsular cataract.

From a clinical point of view of importance to patients, the number of participants who lost vision or needed cataract surgery was substantially the same in the participants taking vitamin/minerals and in the participants taking placebo.

Moreover no harmful side effects were observed in patients on vitamin/minerals.

Because of the qualitatively different effect of vitamins/minerals on the different types of age-related cataract we cannot recommend a regular use of vitamin/minerals for the prevention of cataract.

We are very interested in your opinion on this letter so we invite you to complete the attached questionnaire. Should you need further clarifications on the results of the study or should you wish to know the treatment to which you were assigned please contact Sally Louise Williams, the study manager, at the following telephone number: 0521-980844.

Sally Louise Williams Prof. Giovanni Maraini

CTNS Study Manager CTNS Principal Investigator
