# Supplementary material for: A post-trial survey to assess the impact of dissemination of results and unmasking on participants in a 13-year randomised controlled trial on age-related cataract
Source: Trials. 2011 Jun 14;12:148. doi: 10.1186/1745-6215-12-148 (PMC3136405; doi:10.1186/1745-6215-12-148)
Supplement: Additional file 2 — Post-trial questionnaire 1. Questionnaire to assess patient satisfaction and understanding of the results. [file 1745-6215-12-148-S2.DOC]

**Post-Trial Questionnaire 1**

Enclosed with the letter describing the results of the study, we are sending the following short questionnaire to understand whether you found the information useful and satisfactory.

If you are willing to complete the questionnaire, please give only one answer to each question, and return the questionnaire using the enclosed pre-paid envelope.

Please feel free to express your opinion or not to complete the questionnaire.

All the information you will provide will be considered strictly confidential in agreement with the Privacy Code (D.Lgs. n. 196/2003)

**1. Name**…………………. **2. Surname**………………………………

**3. Date of birth**……………………………………...

**4. Do you think the description of the results of the study is clear?**

□ Very clear

□ Quite clear

□ Not clear

□ Don’t know

**5. Do you think the results of the study are interesting?**

□ Very interesting

□ Quite interesting

□ Not interesting

□ Don’t know

**6. What did you feel when you learned about the results of the study?**

□ Satisfaction

□ Concern

□ Both satisfaction and concern

□ Indifference

□ Don’t know

**7. Do you think it is appropriate to receive the results of the study by letter?**

□ Very appropriate

□ Quite appropriate

□ Not appropriate

□ Don’t know

**8. Are you interested in having further clarifications from the study staff?**

□ Yes

□ No

□ Don’t know

**9. Are you interested in knowing whether you took vitamins and minerals during the study or a placebo?**

□ Yes

□ No

□ Don’t know

**10. During the study how often did you not eat fruit and vegetables or other foods because you thought that the daily study pill contained a sufficient quantity of vitamins and minerals?**

□ Often

□ Sometimes

□ Rarely

□ Never

□ Don’t know

**11. Would you recommend to other persons to take part in a study like CTNS?**

□ Yes, definitely

□ Yes, probably

□ Probably not

□ Definitely not

□ Don’t know
