# Supplementary material for: A post-trial survey to assess the impact of dissemination of results and unmasking on participants in a 13-year randomised controlled trial on age-related cataract
Source: Trials. 2011 Jun 14;12:148. doi: 10.1186/1745-6215-12-148 (PMC3136405; doi:10.1186/1745-6215-12-148)
Supplement: Additional file 3 — Post-trial questionnaire 2. Questionnaire to assess patient understanding of treatment assignment to supplement or placebo and the success of masking. [file 1745-6215-12-148-S3.DOC]

**Post-Trial Questionnaire 2**

**(to be completed by Study Manager or Interviewer at the clinic or on the telephone)**

**1. Name**……………………… **2. Surname**………………………………

**3. Responded to post-trial questionnaire 1:**

□ At home

□ On the telephone

□ In person

□ Did not respond

**4. Responded to post-trial questionnaire 2:**

□ On the telephone

□ In person

□ Did not respond

**5. Do you wish to know whether you took vitamin and minerals during the study or a placebo?**

□ Yes

□ No

□ Don’t know

**6. Evaluation of the Study Manager (or Interviewer):**

The patient understood the randomization process: □ Yes □ No

**7. In your opinion what treatment did you take?**

□ Vitamins and minerals

□ Placebo

□ Don’t know

**DISCLOSURE OF TREATMENT ASSIGNMENT**

**8. Now that you know what treatment you took would you recommend to other persons to take part in a similar study?**

□ Yes, definitely

□ Yes, probably

□ Probably not

□ Definitely not

□ Don’t know
